# Measurement of Underwater Acoustic Energy Radiated by Single Raindrops

**DOI:** 10.3390/s21082687

**Published:** 2021-04-11

**Authors:** Shu Liu, Qi Li, Dajing Shang, Rui Tang, Qingming Zhang

**Affiliations:** 1 Acoustic Science and Technology Laboratory, Harbin Engineering University, Harbin 150001, China; oucshushu@hrbeu.edu.cn (S.L.); liqi@hrbeu.edu.cn (Q.L.); tangrui@hrbeu.edu.cn (R.T.); zhachuan@hrbeu.edu.cn (Q.Z.); 2 Key Laboratory of Marine Information Acquisition and Security (Harbin Engineering University), Ministry of Industry and Information Technology, Harbin 150001, China; 3 College of Underwater Acoustic Engineering, Harbin Engineering University, Harbin 150001, China

**Keywords:** rainfall noise, pressure distribution, frequency distribution, sound energy, energy conversion efficiency

## Abstract

Underwater noise produced by rainfall is an important component of underwater ambient noise. For example, the existence of rainfall noise causes strong disturbances to sonar performance. The underwater noise produced by a single raindrop is the basis of rainfall noise. Therefore, it is necessary to study the associated underwater noise when drops strike the water surface. Previous research focused primarily on the sound pressure and frequency spectrum of underwater noise from single raindrops, but the study on its sound energy is insufficient. The purpose of this paper is to propose a method for predicting the acoustic energy generated by raindrops of any diameter. Here, a formula was derived to calculate the underwater sound energy radiated by single raindrops based on a dipole radiation pattern. A series of experiments were conducted to measure the underwater sound energy in a 15 m × 9 m × 6 m reverberation tank filled with tap water. The analysis of the acoustic energy characteristics and conversion efficiency from kinetic to acoustic energy helped develop the model to predict the average underwater sound energy radiated by single raindrops. Using this model, the total underwater sound energy of all raindrops during a rainfall event can be predicted based on the drop size distribution.

## 1. Introduction

Rain and wind generated acoustic sound from the ocean surface represents the background baseline of ocean ambient noise. Some studies have shown that when present, the acoustic sound generated by rainfall dominates the underwater sound field [1,2,3]. There are two distinct acoustic fields associated with rainfall. The first is associated with drizzle or light rain and is characterized by a broad spectral peak near 15 kHz [2]. The second, associated with heavy rainfall, is highly correlated with the rainfall rate, and is nearly white [3]. The study of rainfall noise is important to marine physics not only to study wind and rainfall over the ocean [4] but also for the use of sonars [5,6,7]. This is also important for biologists when studying the impact of artificially generated sound on marine mammals [8].

There have been some attempts to illustrate the effects of raindrops falling on a water surface. Worthington [9] made the first photographic study. Then, Franz [10], Laville [11] and Buckingham [12] identified two main sources of underwater acoustic sound from a water drop striking the surface: the bubble sound signal as a decaying sinusoid and the initial impact sound signal as a sharp pulse. Franz also observed that the bubble and impact sounds behaved as simple dipole sources near the water surface.

As Franz’s research was limited to large drops striking the water surface, Medwin and Nystuen [13] examined the sound produced by individual water drops over the entire size range of rainfall to ensure the applicability of the results. They showed that the sound spectra of rainfall can be dissected into impact sound or bubble sound within four acoustically distinctive ranges of drop diameters, d. They defined ”minuscule” drops (d < 0.8 mm), “small” drops (0.8 mm ≤ d ≤ 1.1 mm), “mid-size” drops (1.1 mm ≤ d ≤ 2.2 mm) and “large” drops (d > 2.2 mm).

**Figure 1:**
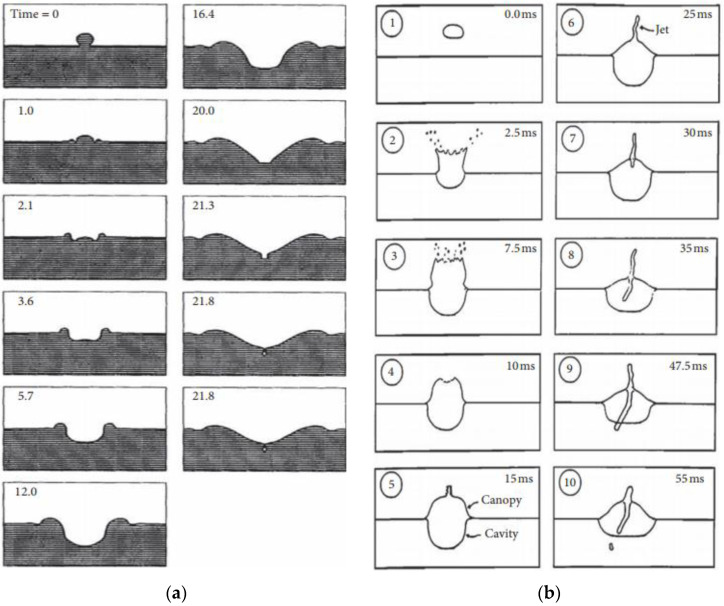
Physical sequences of raindrop splashes: (**a**) small raindrop splash (redrawn from Figure 9 in [19]); (**b**) large raindrop splash (redrawn from Figure 2 in [17]).

There have been several laboratory experiments performed to describe the sound produced by single raindrops [14,15,16,17]. These experiments identified four mechanisms for raindrop-produced sound: (i) initial impact, (ii) type-I bubble, (iii) type-II bubble and (iv) type-III bubble. A bubble-entrapment mechanism has been identified for light rain and drizzle where the pinch-off of the tip from the splash crater is from gravity and surface tension [14,18,19]. The bubbles formed by this mechanism are defined as type-I [13]. The process whereby a type-I bubble forms is shown in Figure 1a. The raindrop hits the water surface at time t = 0, and the bubble is produced at t = 21.8 ms. Larger raindrops are present in heavier rainfall, the process whereby a type-II bubble forms is shown in Figure 1b. A canopy is formed when a spray of droplets is ejected by the upward-moving water mass. At the apex of the canopy, water converges and produces upward- and downward-moving turbulent jets of water. A bubble is entrapped underwater as the downward-moving jet penetrates the crater bottom. Bubbles created from this mechanism are designated as type-II [13]. The “delayed bubbles” occur more than 100 ms after the initial impact of large raindrops and are produced during the re-entry splashes of the corona droplets thrown upward by the initial impact. Bubbles formed from this mechanism are defined as type-III [17].

The bubbles produced by raindrops are the primary source of underwater noise due to rain. The dynamics of underwater bubbles have long been of considerable interest because of their importance in various physical and engineering problems, including cavitation on ship propellers [20,21,22], underwater explosions [23,24,25] and ultrasonic cleaning [26,27,28].

A detailed dynamic associated with bubble bursting was presented [29]. Using experimental observations and numerical simulations, Prosperetti [30] gave a brief history and explanation for the impact of drops on liquid surfaces and the underwater noise from rain. The strong bubble interactions and bursting behaviors near a free surface have been studied numerically with a compressible two-phase flow solver [31]. Heindsmann [32] made the first attempt to describe underwater noise spectra produced by natural rainfall. He found that during the heaviest rainfall, the sound pressure spectrum level was approximately constant at 77 dB (ref: 1 μPa) from below 1 to above 10 kHz.

The underwater sound produced by rainfall has unique characteristics. The measurement for this type of sound signal can be used to predict and measure rainfall. Field measurements of subtropical rainfall have been used to demonstrate the forward (predicting the sound field given the rainfall drop size distribution) and the inverse (estimating the rainfall drop size distribution given the sound field) problems [33]. One of the first efforts was to make acoustic-based geophysical measurements from an ocean surface mooring. The acoustic data were interpreted to provide detection, classification and quantification of rainfall. A modified acoustic rainfall algorithm was then proposed [34]. A semi-empirical model was also proposed to predict the ambient sound spectra from 0.5–50 kHz at rainfall rates from 2–200 mm/h and wind speeds from 2–14 m/s [35].

There has been significant research on the physical properties of bubbles but little research [36] on the acoustic energy radiated by a single bubble as produced from a raindrop. There have been no studies on the acoustic energy radiated by the initial impact produced from a raindrop. Therefore, there is no systematic study of how much acoustic energy a single raindrop radiates. To address this important research gap, a formula was derived to calculate the underwater sound energy of raindrop radiation, which includes the impact and bubble energies. The purpose of this paper is to propose a method for predicting the acoustic energy generated by raindrops of any diameter. For this purpose, a series of experiments were performed to measure the underwater sound energy radiated by single raindrops in a reverberation tank, and the associated characteristics were analyzed. The conversion efficiency from kinetic to acoustic energy was calculated. Based on the theoretical and experimental results, a model was proposed to predict the underwater sound energy radiated from single raindrops. By analyzing the conversion efficiency, the average underwater sound energy radiated by single raindrops of any diameter can be predicted. The results show that the prediction model is effective as the total underwater sound energy of all raindrops during rainfall can be calculated using the model. Once the average sound energy is known, the total underwater sound energy during a rainfall event can be predicted based on the drop size distribution. The model can also be used to predict the underwater sound energy radiated from other liquids, such as waterfalls and water discharged from a ship’s outlet, when hitting the water surface.

## 2. Methods

### 2.1. Theory

#### 2.1.1. Underwater Acoustic Energy

**Figure 2:**
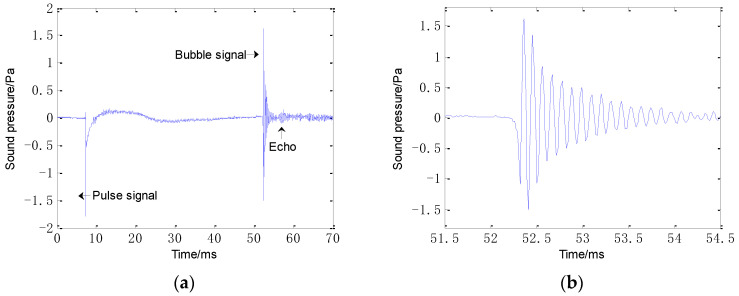
Acoustic signal produced from a 3.9 mm raindrop: (**a**) entire signal and (**b**) sinusoidally damped bubble signal.

The sound spectra of raindrops can be dissected into impact and bubble sounds. Figure 2 shows the acoustic signal produced by a raindrop with a 3.9-mm diameter. In Figure 2a, the signal consists of the initial pulse signal and bubble signal. The sinusoidally damped bubble signal is shown in Figure 2b with an appropriate amplitude amplification.

The bubble produced from a single raindrop is usually assumed to be spherical. The resonance frequency of the bubble is given by: [37]

(1) $$f_{0}=\frac{1}{\pi d}\sqrt{\frac{3\gamma P_{0}}{\rho }}\;,$$

where d is the bubble diameter, $P_{0}$ is the ambient pressure, ρ = 998 kg/m^3^ is the water density and γ is the ratio of the specific heat of air (γ = 1.4).

**Figure 3:**
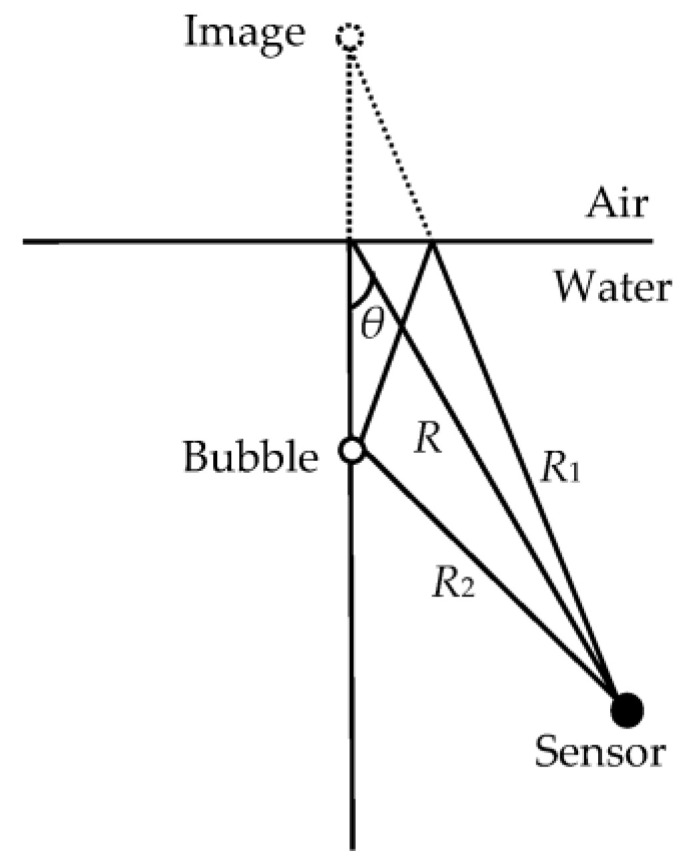
Sound-radiation model of a bubble near the air-water interface.

A near-surface oscillating bubble forms a phase-reversed image at the smooth, reflecting water-air interface and behaves as a dipole. The far-field theoretical radiation pressure of the bubble acts as a dipole [38] and is proportional to cosθ, where θ is the angle from the direction perpendicular to the water surface. This sound-radiation model for bubbles is illustrated in Figure 3, where *R*, *R*_1_ and *R*_2_ are the distances from the point of impact, the image and the bubble to the sensor, respectively.

The energy radiated by a surface dipole [17] is given by:

(2) $$E_{b}=\frac{2\pi R^{2}}{3\rho c}\int p_{b}^{2}\left(t\right)dt,$$

where *R* is the distance from the point of impact to the hydrophone, c = 1480 m/s is the speed of sound in water and$\;p_{b}\left(t\right)$ is the axial acoustic pressure of the bubble signal at the range *R*. The acoustic energy of the bubble can be calculated using Equation (2) once $\;p_{b}\left(t\right)$ has been measured.

Franz [10] noted that the impact sound of a drop behaves as a simple dipole source near the water surface. The acoustic energy of the initial pulse signal can be calculated using:

(3) $$E_{p}=\frac{2\pi R^{2}}{3\rho c}\int p_{p}^{2}\left(t\right)dt,$$

where $p_{p}\left(t\right)$ is the axial acoustic pressure of the initial pulse signal at the range *R*. The underwater acoustic energy radiated by a single bubble produced from a raindrop is the sum of $E_{b}$ and $E_{p}$ as:

(4) $$E=E_{b}+E_{p}.$$

#### 2.1.2. Kinetic Energy

Mechanics demonstrated that the velocity of a raindrop in air tended asymptotically towards a limit defined by its terminal velocity. The terminal velocity for raindrops in air has been studied by many investigators [39,40,41,42]. However, it is difficult to accurately calculate the velocity of raindrops, and there are several different but consistent empirical formulas to this end. The formula proposed by Mou [41] was used here. For relatively small drops (d < 0.5 mm), the terminal velocity can be calculated using Stokes drag formula as

(5) $$v=2985d^{2}.$$

For average drops (0.5 mm ≤d≤ 1.9 mm), the terminal velocity can be calculated as:

(6) $$v=0.496\times 10^{n},$$

where:

(7) $$n=\sqrt{28.32+6.524\mathrm{lg}\left(0.1d\right)-{\left(\mathrm{lg}0.1d\right)}^{2}}-3.665,$$

where lg means take the common logarithm.

For large drops (1.9 mm <d< 5 mm), the terminal velocity can be calculated as:

(8) $$v=\left(17.2-0.844d\right)\cdot \sqrt{0.1d}.$$

The raindrops become unstable and explode when their diameter exceeds 5 mm. In the above formula, the diameter is in mm while the velocity is in m/s. Once the terminal velocity of a raindrop is given, its kinetic energy can be calculated as:

(9) $$E_{k}=\frac{1}{2}mv^{2},$$

where m is the raindrop mass.

### 2.2. Experiment

**Figure 4:**
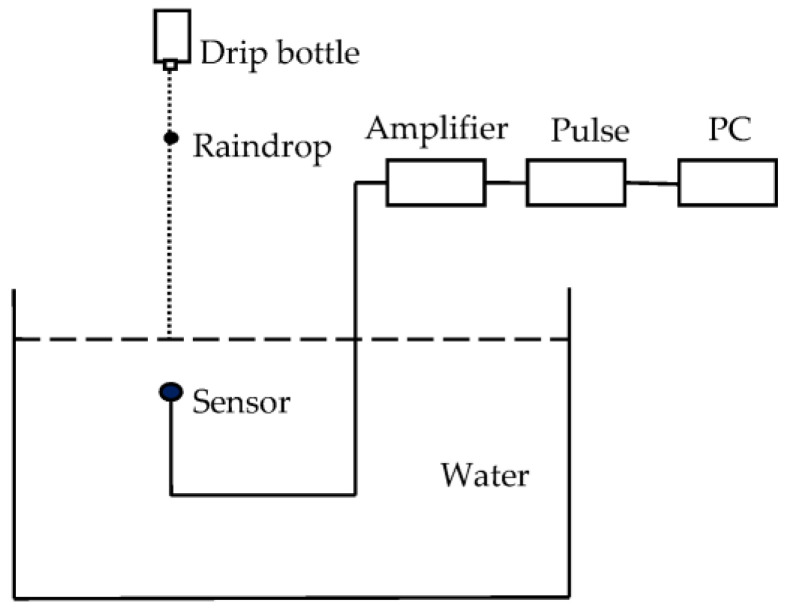
System used to measure sound signal generated by drops.

To study the underwater acoustic energy radiated from a single raindrop, a series of experiments was conducted in a reverberation pool (15 m × 9 m × 6 m) filled with tap water. The temperature of the water was 20 °C and the temperature of the air was 18.5 °C. The system used to measure the sound signal generated by raindrops is shown in Figure 4. There were four acoustically distinct ranges of drop diameter *d* defined as (i) minuscule drops (*d* ≤ 0.8 mm), (ii) small drops (0.8 mm < *d* ≤ 1.1 mm), (iii) mid-size drops (1.1 mm < *d* ≤ 2.2 mm) and (iv) large drops (*d* > 2.2 mm). We used a standard intravenous drip bottle with needles of different calibers to generate drops with five different diameters of 0.7, 0.9, 1.5, 2.5 and 3.9 mm. The height of the drop bottle was 6 m. At this height, each drop struck the water surface at the terminal velocity of raindrops in natural rainfall. A hydrophone (type 8103; Brüel & Kjær, Skodsborgvej 307 DK-2850 Nærum, Denmark) was suspended 20 cm below the impact point. At this distance, the effects of reflected sounds are avoided. The first echo is calculated as impacting the hydrophone after approximately 7.8 ms (t=2×(6−0.2)/1480≈ 0.0078 s = 7.8 ms). The duration of the acoustic signal from the bubble produced by raindrops was mostly less than 2 ms [36]. As shown in Figure 2, the echo and direct sound are distinguished, where the echo is much smaller. In general, the pool used in the experiments was considered as a reverberation pool. The acoustic pressure signal was transient and easily contaminated by tank reverberations. However, the pool was sufficiently large to eliminate these effects. The hydrophone was connected to a pulse collector (type 3560E; Brüel & Kjær, Skodsborgvej 307 DK-2850 Nærum, Denmark) and the signal was stored in a PC.

## 3. Results and Discussion

### 3.1. Probability

Water drops with five different diameters were generated in the experiments. As shown in Table 1, the 0.7 mm (minuscule) drop produced only a weak initial impact sound. The 0.9 mm (small) drop produced a weak initial impact sound and a type-I damped bubble with a 100% probability. The 1.5 mm (mid-size) drop produced only a weak initial impact sound. The 2.5 mm (large) and 3.9 mm (large) drops both produced initial impact sounds along with type-II and III damped bubbles. The 2.5 (3.9) mm drop had a 27.6% (57.9%) probability of producing a type-II bubble and a 34.0% (23.4%) probability of producing a type-III bubble.

Li et al. described why the 1.5 mm drop produced only impact noise and no bubble whereas the 0.9 and 2.5 mm drops both produce bubbles [36]. Compared with the underwater acoustic energy of the bubble sound, the energy of the weak impact sound produced by water drops at 0.7, 0.9 and 1.5 mm was too small to be negligible. Except for large raindrops, the underwater acoustic energy of the initial impact signal produced by other raindrops is ignored in this paper.

### 3.2. Peak Axial Sound Pressure

**Figure 5:**
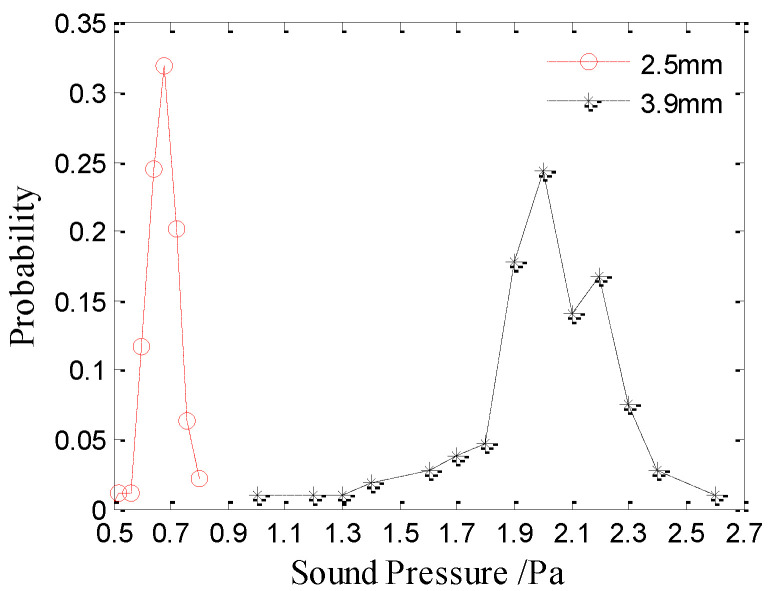
Sound amplitude distributions of the initial impact noise generated by raindrops with diameters of 3.9 mm (black asterisks) and 2.5 mm (red circles).

The underwater acoustic signal collected from the pulse collector was converted into sound pressure at 1 m on the axis based on the dipole radiation pattern. The peak axial sound pressure of the bubble and the initial impact at 1 m are considered here. The peak axial sound pressure values mentioned in this paper are all the sound pressure at 1 m distance on the axis. According to the statistical results, the peak axial sound pressure of the initial impact noise generated by raindrops at 3.9 and 2.5 mm is 2.02 and 0.65 Pa, respectively. The amplitude distributions of the initial impact noise are shown in Figure 5. The results show that the peak axial sound pressures of the initial impact noise generated by raindrops at 3.9 and 2.5 mm is approximately a Gaussian distribution with means of 2.0 and 0.68 Pa, respectively. The peak axial sound pressure of the initial impact noise increases with the raindrop diameter.

**Figure 6:**
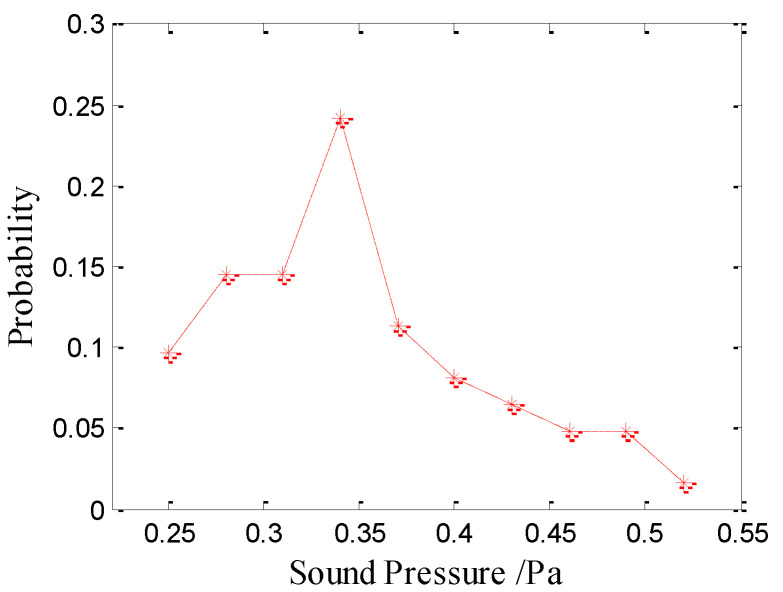
Amplitude distribution of the type-I bubble noise produced by raindrops with diameters of 0.9 mm.

The peak axial sound pressures of type-I bubble noise produced by raindrops at 0.9 mm is approximately Poisson distribution with a mean of 0.34 Pa. The average peak axial sound pressure for the type-I bubble noise is 0.36 Pa. Figure 6 shows the amplitude distribution of the type-I bubble noise produced from 0.9 mm raindrops.

**Figure 7:**
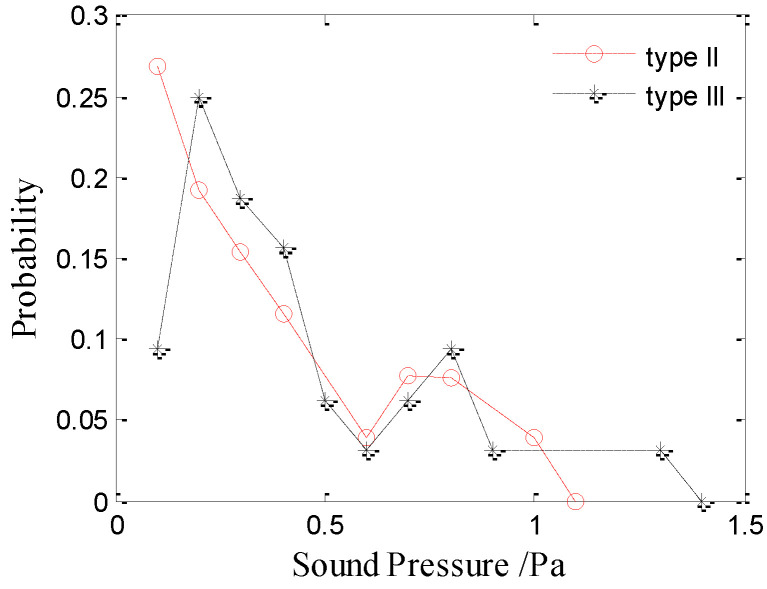
Amplitude distributions of the type-II and type-III bubble noise produced by raindrops with a diameter of 2.5 mm.

**Figure 8:**
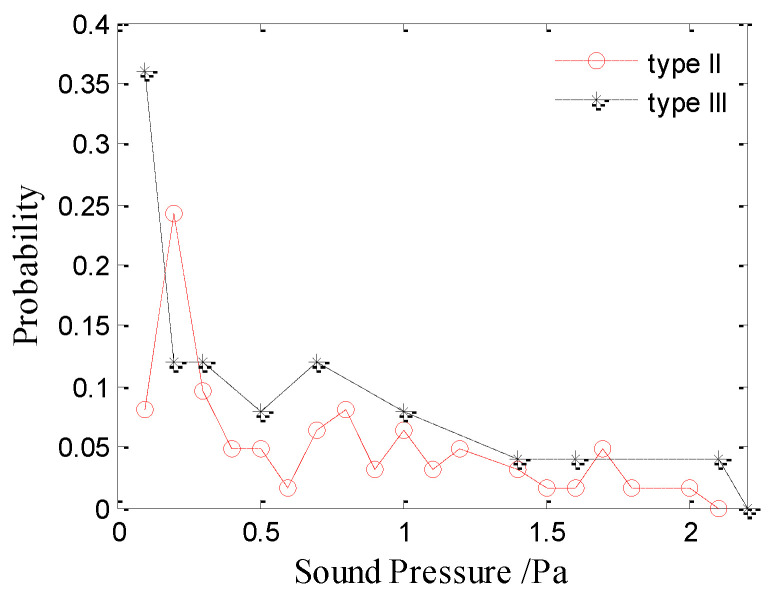
Amplitude distributions of the type-II and type-III bubble noise produced by raindrops with a diameter of 3.9 mm.

Figure 7 and Figure 8 show the amplitude distributions of the type-II and type-III bubble noise produced by raindrops at 2.5 mm and 3.9 mm, respectively. The results show that the amplitude distributions of the type-II and III bubble noise are random and dispersive, whereas the distribution of the type-I bubble noise is relatively concentrated. As shown in Figure 7 and Figure 8, there is no significant difference between the amplitude distributions of the type-II and type-III bubble noise produced by raindrops of 2.5 or 3.9 mm. Therefore, the sound pressure amplitude of the type-II and type-III bubbles are discussed together.

**Figure 9:**
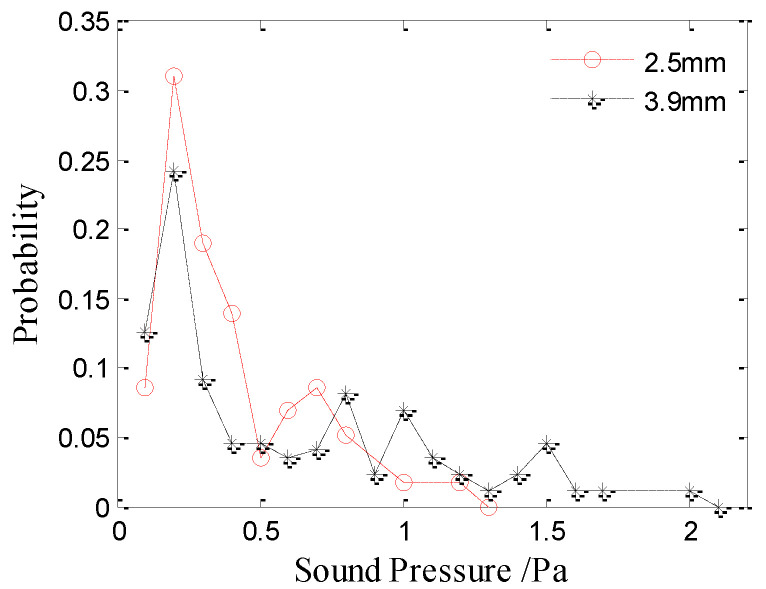
Amplitude distributions of the bubble noise produced by raindrops with diameters of 2.5 and 3.9 mm.

The amplitude distributions of bubble noise (type-II and type-III included) produced by raindrops at 2.5 mm and 3.9 mm are shown in Figure 9, which are random and dispersive. Interestingly, the curves have a peak at 0.2 Pa in Figure 9. As the raindrop diameter increases, more bubbles with a higher sound pressure are produced.

### 3.3. Frequency Range

The frequency range of the initial impact and bubble sounds can be obtained by performing a Fourier transform on the sound pressure signal. The results show that the spectrum for the single initial impact sound is broadband, and the frequency range of the impact sounds produced by different raindrops of the same size is nearly the same. The frequency of the initial impact sounds generated by raindrops at 2.5 and 3.9 mm range from 0–20 kHz and 0–30 kHz, respectively.

**Figure 10:**
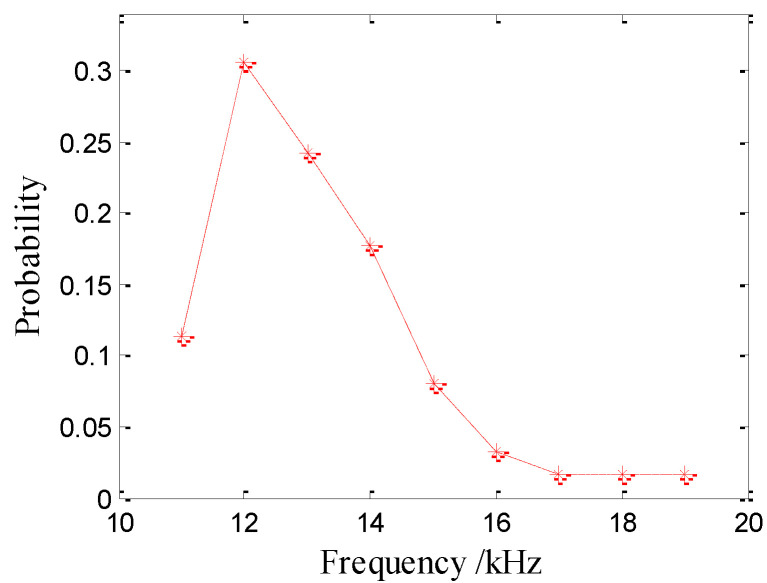
Frequency distributions of the bubble noise produced by raindrops with a diameter of 0.9 mm.

Even though the spectrum of the signal radiated from a sinusoidally damped bubble is simple, the frequency distribution of many bubbles of the same size is relatively complex. The bubble resonance frequencies are observed for 263 water drops (62 at 0.9 mm, 94 at 2.5 mm and 107 at 3.9 mm). The probability distributions of the bubble sounds are shown in Table 1. The peak resonance frequency of bubbles generated by 0.9-mm drops is 12 kHz and ranges from 11–19 kHz (see Figure 10), where the cumulative percentage between 11–15 kHz is 92%.

**Figure 11:**
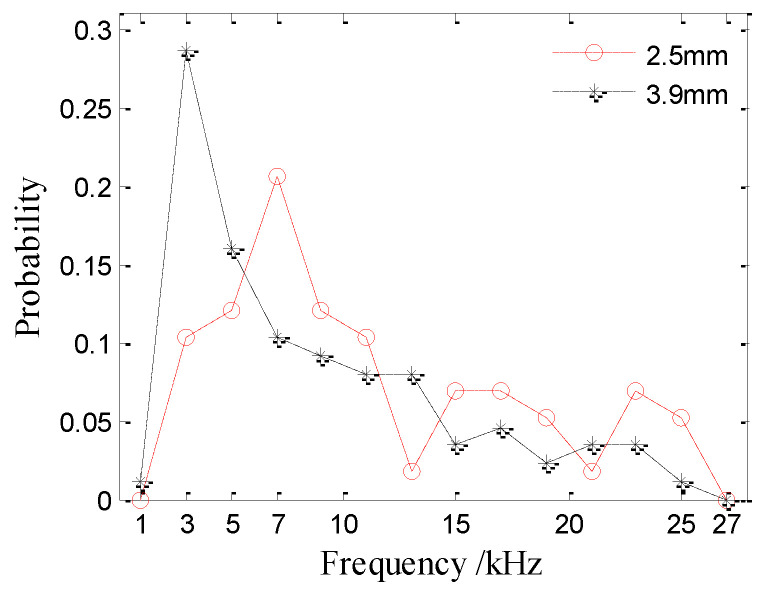
Frequency distributions of the bubble noise produced by raindrops with diameters of 2.5 and 3.9 mm.

The frequency distributions of the bubble noise produced by raindrops at 2.5 mm and 3.9 mm are shown in Figure 11. The peak resonance frequency of bubbles generated by 2.5 (3.9) mm drops is 5 (3) kHz with a range from 2.4–25.4 (1.9–25.0) kHz. As the raindrop diameter increases, more bubbles with a lower resonance frequency are produced. The cumulative percentage for the 2.5 mm drops from 1–5 kHz is 22.4%, and that for 3.9 mm drops from 1–5 kHz is 46.0%.

Of note, the diameter of the largest bubble produced by a raindrop of a given size is approximately the same as the diameter of the raindrop (see Table 2). As shown in Table 2, d is the diameter of the raindrop, $f_{m}$ is the lowest resonance frequency of the bubbles generated by the corresponding raindrop and D is the diameter of the bubble calculated using Equation (1). The raindrop size determines the lowest cut-off resonance frequency of the produced bubbles.

### 3.4. Acoustic Energy

**Figure 12:**
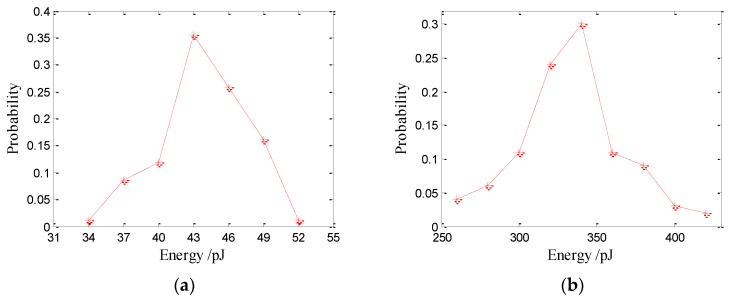
Underwater acoustic energy distributions of the initial impact noise produced by large raindrops: (**a**) 2.5 and (**b**) 3.9 mm.

The acoustic energy of the bubble sound produced by raindrops can be calculated using Equation (2), while the acoustic energy of the initial impact sound is calculated from Equation (3). The underwater acoustic energy distributions of the initial impact noise are shown in Figure 12. The results suggest that the energies of the initial impact noise generated by raindrops at 2.5 and 3.9 mm approximately follow Gaussian distributions with means of 43 pJ (1 pJ = 10^−12^ J) and 340 pJ, respectively. The underwater acoustic energy of the initial impact noise increases with the raindrop diameter. The average acoustic energy of the initial impact noise generated by raindrops at 2.5 and 3.9 mm is 44.6 and 350 pJ, respectively. The energy of the initial impact noise generated by 2.5 mm raindrops ranges from 34–52 pJ. The energy of the initial impact noise generated by 3.9 mm raindrops ranges from 260–420 pJ.

**Figure 13:**
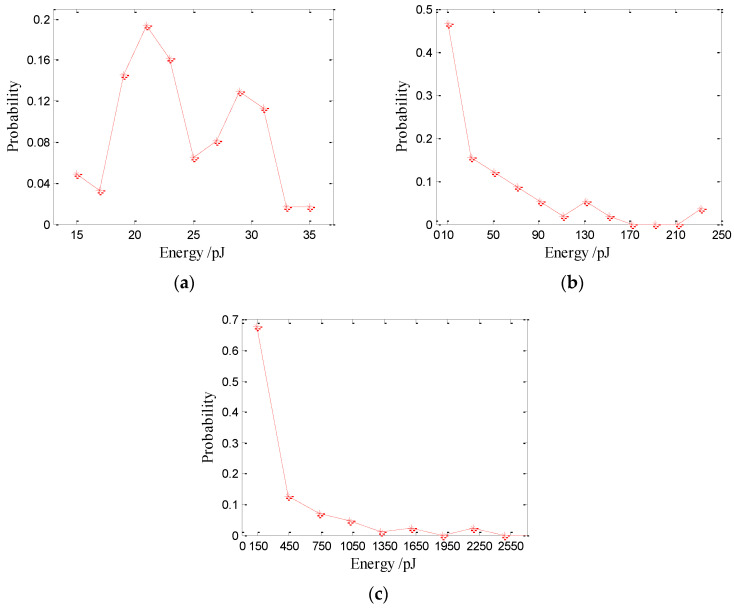
Underwater acoustic energy distributions of bubble noise produced by raindrops: (**a**) 0.9, (**b**) 2.5 and (**c**) 3.9 mm.

The underwater acoustic energy distributions of bubble noise produced by raindrops are shown in Figure 13. The peak acoustic energy for bubbles generated by 0.9 mm drops is 21 pJ with a range of 14.6–35 pJ (see Figure 13a), while the average acoustic energy of bubbles generated by 0.9 mm drops is 24.1 pJ.

The sound pressure, resonance frequency and acoustic energy distributions of the bubble noise generated by 0.9 mm drops are relatively concentrated. Whereas the energy distributions of the bubble noise generated by 2.5 and 3.9 mm drops are dispersive and random. The energy of the bubble noise generated by 2.5 (3.9) mm drops ranges from 0.4–243 (0.5–2200) pJ. With an increased drop size, the acoustic energy produced by raindrops covers a wider range. Table 3 shows the average energy of the initial impact sound and bubble sound produced by drops of different diameters. The $\bar{E_{p}}$ and $\bar{E_{I}}$ are the average energies of the initial impact and type-I bubbles, respectively, and $\bar{E_{\mathrm{II}}}$ and $\bar{E_{\mathrm{III}}}$ are the average energies of the type-II and type-III bubbles, respectively.

**Figure 14:**
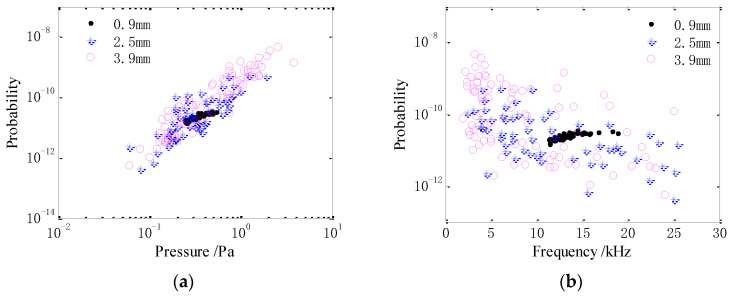
Acoustic energy of the bubble noise produced by raindrops versus the (**a**) sound pressure and (**b**) resonance frequency.

Figure 14 shows the acoustic energy of the bubble noise produced by raindrops versus the sound pressure and resonance frequency. As shown in Figure 14a, the sound energy for drops of different diameters increases with the sound pressure. As the drop diameter increases, more bubbles with a higher sound pressure are produced. With an increased drop size, the range of the sound pressure amplitude and energy of the bubble noise becomes wider. As shown in Figure 14b, the sound energy of bubbles produced by 2.5 and 3.9 mm drops decreases with the resonance frequency. Whereas the sound energy of bubbles produced by 0.9 mm drops increases with the resonance frequency. As the drop diameter increases, more bubbles with lower resonance frequencies are produced. With an increased drop size, the range of the resonance frequency and sound energy of the bubble noise widen.

### 3.5. Conversion Efficiency

When a raindrop hits the water surface at its terminal velocity, its kinetic energy can be calculated using Equation (9). The energy of the underwater acoustic noise produced from a raindrop can be calculated using Equation (4). The efficiency of the kinetic energy conversion from the droplet to sound energy radiated into the water is the ratio of the sound to kinetic energies. Not every raindrop produces bubbles and raindrops of the same size do not produce bubbles with the same acoustic energies. Therefore, for a single raindrop, the efficiency of the sound energy conversion is of no practical significance. However, it makes sense to calculate the average kinetic energy conversion efficiency to sound energy for raindrops of the same size, which is defined as:

(10) $$\eta =\frac{\sum E_{b}\left(i\right)+\sum E_{p}\left(i\right)}{\sum E_{k}\left(i\right)}\;,$$

where $E_{b}\left(i\right)$ and $E_{p}\left(i\right)$ are the bubble sound energy and initial impact energy radiated by the *i*th raindrop of the same diameter, respectively, and $E_{k}\left(i\right)$ is the kinetic energy of raindrops of the same diameter.

The efficiency of kinetic energy conversion of the droplet to sound energy radiated into the water is shown in Table 4. The $E_{p}$ and $E_{b}$ are the total initial impact and bubble energies radiated by the raindrops, and $E_{k}$ is the total kinetic energy of the raindrops. There are 62 drops at 0.9 mm, 94 drops at 2.5 mm and 107 drops at 3.9 mm.

For large raindrops of the same diameters, the total initial impact energy and the total bubble energy are nearly the same. The conversion efficiency of the 3.9 mm raindrops is greater than that of the 2.5 mm raindrops. As the raindrop diameter increases, more bubbles with lower resonance frequencies and greater sound energy are produced. For small raindrops, only the bubble sound energy contributes to underwater noise. The efficiency of conversion of kinetic energy from the raindrop to sound energy radiated into the water is 1.04 × 10^−^^5^. In other words, only around 10^−5^ of the kinetic energy of the drop goes into the total acoustic radiation. The efficiency of conversion of small raindrops is much greater than that of the large raindrops for two reasons: (i) bubbles are created 100% of the time for small raindrops and (ii) the kinetic energy of small raindrops is much less than that of large raindrops.

### 3.6. Proposed Model

As noted in Section 3.1, only small (0.8 mm ≤d≤ 1.1 mm) and large (d> 2.2 mm) drops produce distinct underwater acoustic noise. A model is proposed based on the above study to predict the average underwater sound energy radiated by single raindrops.

The average underwater sound energy $E_{\mathrm{as}}$ radiated by small raindrops is expressed as:

(11) $$E_{\mathrm{as}}=\eta_{s}E_{\mathrm{ks}},$$

where $\eta_{s}≅1\times 10^{-5}$ is the kinetic energy efficiency of conversion for small raindrops to sound energy radiated into the water, and $E_{\mathrm{ks}}$ is the kinetic energy of a small raindrop.

The average underwater sound energy $E_{\mathrm{al}}$ radiated from a large raindrop consists of the bubble and initial impact sound energies and is expressed as:

(12) $$E_{\mathrm{al}}=E_{\mathrm{pl}}+E_{\mathrm{bl}}=(\eta_{\mathrm{pl}}+\eta_{\mathrm{bl}})E_{\mathrm{kl}},$$

where $E_{\mathrm{pl}}$ and $E_{\mathrm{bl}}$ are the initial impact and bubble sound energies radiated from a large raindrop, respectively, and $\eta_{\mathrm{pl}}$ and $\eta_{\mathrm{bl}}$ are the kinetic energy efficiencies of conversion for large raindrops to the initial impact and bubble sound energies radiated into the water, respectively. From the data in Table 4, the $\eta_{\mathrm{pl}}$ and $\eta_{\mathrm{bl}}$ are approximately equal:

(13) $$\eta_{\mathrm{pl}}≅\eta_{\mathrm{bl}}≅d\times 10^{-7}\;.$$

Therefore, for any raindrop of diameter d, the average underwater acoustic energy $E_{a}$ radiated from it can be expressed as:

(14) $$E_{a}=\eta E_{k}\;,$$

where η is the kinetic energy efficiency of conversion for raindrops to sound energy. The conversion efficiency is shown in Table 5.

### 3.7. Discussion

Once the average underwater acoustic energy for any type of single raindrop has been calculated, the total underwater sound energy for all raindrops during a rainfall event can be predicted based on the drop size distribution as generalized from the empirical Marshall-Palmer expression [43]. In this expression, the total number of raindrops with diameter d in a realistic rainfall is:

(15) $$n\left(d\right)=N_{0}e^{-\Lambda d},$$

where $N_{0}$ is a constant and Λ is a function of rainfall rate R.

When the rainfall rate R is measured, the number of raindrops with diameter d can be calculated. The total underwater sound energy $E_{d}$ for raindrops with diameter d can be expressed by using the average underwater acoustic energy $E_{\mathrm{ad}}$:

(16) $$E_{d}=n\left(d\right)E_{\mathrm{ad}}=n\left(d\right)\eta_{d}E_{\mathrm{kd}},\;$$

where $\eta_{d}$ is the kinetic energy efficiency of conversion for raindrops with diameter d to sound energy and $E_{\mathrm{kd}}$ is its kinetic energy. The total underwater sound energy of all types of raindrops during a rainfall event is:

(17) $$E_{R}=\underset{d}{\sum }E_{d}=\underset{d}{\sum }n\left(d\right)\eta_{d}E_{\mathrm{kd}}.\;$$

By using the conversion efficiency proposed in Section 3.6, the total underwater sound energy of all raindrops during a rainfall event can be predicted.

On the other hand, by studying the correlation between different rainfall rate and corresponding total underwater sound energy, it is possible to invert rainfall rate by measuring underwater sound energy. This may be one of our next research goals.

The model has implications to predict underwater sound energy radiated from other liquids, such as waterfalls and water discharged from ships. If the kinetic energy and the kinetic energy efficiency of conversion to sound energy of these liquids have been analyzed, the total underwater sound energy from these scenarios can be predicted.

## 4. Conclusions

A series of experiments were conducted to measure the underwater acoustic noise produced by a raindrop when it falls onto a planar water surface at its terminal velocity. The acoustic characteristics (including the sound pressure, frequency and sound energy) from the initial impact and bubble sounds were analyzed in detail. The main findings of this study are as follows. Small raindrops produce a type-I damped bubble with a 100% probability. The peak axial sound pressures of bubble noise produced by small raindrops are approximately Poisson distributions. The sound pressure, resonance frequency and acoustic energy distributions of the bubble noise generated by small raindrops are relatively concentrated. The kinetic energy efficiency of conversion for the raindrop to sound energy radiated into the water is $1\times 10^{-5}$.

Large raindrops produce an initial impact sound followed by type-II and III damped bubbles with certain probabilities. The peak axial sound pressures and acoustic energies for the initial impact noise generated by large raindrops are approximately Gaussian distributions. The sound pressure, resonance frequency and acoustic energy distributions of the bubble noise generated by large raindrops are random and dispersive. As the raindrop diameter increases, more bubbles with a higher sound pressure, lower resonance frequency and greater acoustic energy are produced. For large raindrops of the same diameter, the total initial impact energy and total bubble energy are nearly the same. For large raindrops of diameter d, the kinetic energy efficiency of conversion for the raindrop to sound energy radiated into the water is $2d\times 10^{-7}$.

A model was proposed to predict the average underwater sound energy radiated by single raindrops. This model showed that the total underwater sound energy of all raindrops during a rainfall event can be predicted based on the drop size distributions. The model has implications to predict the underwater sound energy radiated from other liquids when they hit the water surface at a certain velocity.

## Figures and Tables

**Table 1:** Probability of the initial impact sound and the subsequent bubble sound.

d [mm]	Drop Type	Number of Tests	Impact	Type-I Bubble	Type-II Bubble	Type-III Bubble
0.7	Minuscule	50	weak, 100%	none	none	none
0.9	Small	62	weak, 100%	100%	none	none
1.5	Mid-size	50	weak, 100%	none	none	none
2.5	Large	94	strong, 100%	none	27.6%	34.0%
3.9	Large	107	strong, 100%	none	57.9%	23.4%

**Table 2:** Diameter of the largest bubbles produced by raindrops.

d [mm]	$f_{m}\;[\mathrm{kHz}]$	D
0.9	11.3	0.6
2.5	2.4	2.7
3.9	1.9	3.5

**Table 3:** Average energy of the initial impact and bubble sounds produced by raindrops.

d [mm]	$\bar{E_{p}}\;[\mathrm{pJ}]$	$\bar{E_{I}}\;[\mathrm{pJ}]$	$\bar{E_{\mathrm{II}}}\;[\mathrm{pJ}]$	$\bar{E_{\mathrm{III}}}\;[\mathrm{pJ}]$
0.9	-	24.1	-	-
2.5	44.6	-	56.9	79.3
3.9	350	-	316	574

**Table 4:** Conversion efficiency of kinetic energy into sound energy.

d [mm]	$E_{p}\;[J]$	$E_{b}\;[J]$	$E_{k}\;[J]$	$\eta_{p}$	$\eta_{b}$
0.9	-	1.49 × 10^−9^	1.43 × 10^−4^	-	1.04 × 10^−5^
2.5	4.19 × 10^−9^	4.02 × 10^−9^	1.80 × 10^−2^	2.33 × 10^−7^	2.23 × 10^−7^
3.9	3.75 × 10^−8^	3.40 × 10^−8^	9.21 × 10^−2^	4.07 × 10^−7^	3.69 × 10^−7^

**Table 5:** Value of the conversion efficiency η for different drop sizes.

Drops Size	*η*
Minuscule	0
Small	$1\times 10^{-5}$
Mid-size	0
Large	$2d\times 10^{-7}$

## Data Availability

The data that support the findings of this study are available from the corresponding author upon reasonable request.
